# Synthesis of polydicyclopentadiene using the Cp_2_TiCl_2_/Et_2_AlCl catalytic system and thin-layer oxidation of the polymer in air

**DOI:** 10.3762/bjoc.15.69

**Published:** 2019-03-20

**Authors:** Zhargolma B Bazarova, Ludmila S Soroka, Alex A Lyapkov, Мekhman S Yusubov, Francis Verpoort

**Affiliations:** 1 National Research Tomsk Polytechnic University, Tomsk, 634050, Russian Federation; 2 Laboratory of Organometallics, Catalysis and Ordered Materials, State Key Laboratory of Advanced Technology for Materials Synthesis and Processing, Wuhan University of Technology, Wuhan, 430070, China; 3 College of Arts and Sciences, Khalifa University of Science and Technology, PO Box 127788, Abu Dhabi, UAE; 4 Ghent University, Global Campus Songdo, 119 Songdomunhwa-Ro, Yeonsu-Gu, Incheon 406-840, South Korea

**Keywords:** bis(cyclopentadienyl)titanium dichloride, cationic polymerization, oxidation, polydicyclopentadiene, thin layers

## Abstract

The polymerization process of dicyclopentadiene using a multicomponent catalytic system based on bis(cyclopentadienyl)titanium dichloride and diethylaluminum chloride was studied. It was demonstrated that the application of an excess of the aluminum component leads to the formation of stable charged complexes of blue discoloration, which initiate cationic polymerization of dicyclopentadiene. Unstabilized thin layers of obtained polydicyclopentadiene undergo oxidation and structuring under atmospheric oxygen. Oxidation of polydicyclopentadiene films in air occurs slowly during several weeks and can be determined by the increase of carbonyl and hydroxyl adsorption bands in infrared spectra. Along with oxidation, cross-linking processes occur in polymers, which lead to a change in physical parameters of the layers, and more precisely to a decrease in the permeability of atmospheric oxygen through the layers. Consequently, this leads to the transition of the oxidation from a kinetic mode into a diffusive mode. Such structural changes do not occur in a polymer that was stabilized by adding an antioxidant.

## Introduction

Currently, polymerization of dicyclopentadiene and norbornene derivatives applying various catalyst systems is of great interest [1–7]. Dicyclopentadiene (DCPD) is a secondary product of the ethylene and propylene production and is used as a monomer to obtain a polymer with particular properties – polydicyclopentadiene (PDCPD) [8–9]. Cationic polymerization of DCPD takes place with metal-halide-based catalyst systems and organometallic compounds. A number of scientific reports were dedicated to the investigation of DCPD polymerization based on these systems [10–11]. One of the drawbacks of these catalyst systems is the “excessive hardness” of the system viz. HSAB theory leading to the formation of cross-linked structures and gelation of the system. Substitution of chlorine atoms in the catalyst structure with organic ligands allows reducing of the hardness of the systems and contributes to the generation of products having a linear structure. To realize this, the usage of a catalyst component bearing already organic ligands in its structure – bis(cyclopentadienyl)titanium dichloride (Cp_2_TiCl_2_) is proposed.

Polymers based on DCPD, obtained by cationic polymerization, are characterized by certain disadvantages. They have a low molecular weight, a fairly rigid structure of the polymer chains due to crosslinking processes occurring during polymerization. In addition, DCPD polymers obtained from "hard" catalytic systems, such as TiCl_4_, SnCl_4_, etc., are easily susceptible to oxidation. Catalytic systems which are less "hard" can overcome these disadvantages to some extent.

The aim of this study is to investigate the interaction between Cp_2_TiCl_2_ and diethylaluminum chloride (AlEt_2_Cl) in toluene which results in the formation of a complex, active for the DCPD polymerization. Additionally, optimization of the ratio between the two compounds of the catalyst system was performed using electron spectroscopy. Furthermore, the DCPD polymerization in toluene was investigated using the optimized catalyst system, and also the dynamics of the structural transformations occurring in thin layers of PDCPD during oxidation in air.

Polymers obtained during the dicyclopentadiene polymerization under these conditions are well soluble in aromatic and chlorinated solvents, and from these solutions, smooth transparent films can be produced. However, the surface of PDCPD loses its transparency and becomes dark as a function of time when stored in air. This is attributed to the formation of crosslinking in the polymer structure and oxidation of unsaturated bonds, which are excessively present in the polymer structure [12–14].

Oxidation of thin PDCPD films in air occurs slowly and is observable by the intensity increase of vibrational bands deriving from carbonyl and hydroxy groups in the infrared spectra of the polymers. More specifically, an intensity increase of the wide band at 3400 cm^−1^ is observed, which is assigned to vibrations of hydroxy groups located near various carbon atoms in the main polymer chain. Apart from this, the intensity of the bending vibrations of carboxyl groups at 1700 cm^−1^ and of ether groups at 1030–1080 cm^−1^ increases as well.

## Results and Discussion

### Study of the complex formation between Cp_2_TiCl_2_ and AlEt_2_Cl

**Figure 1:**
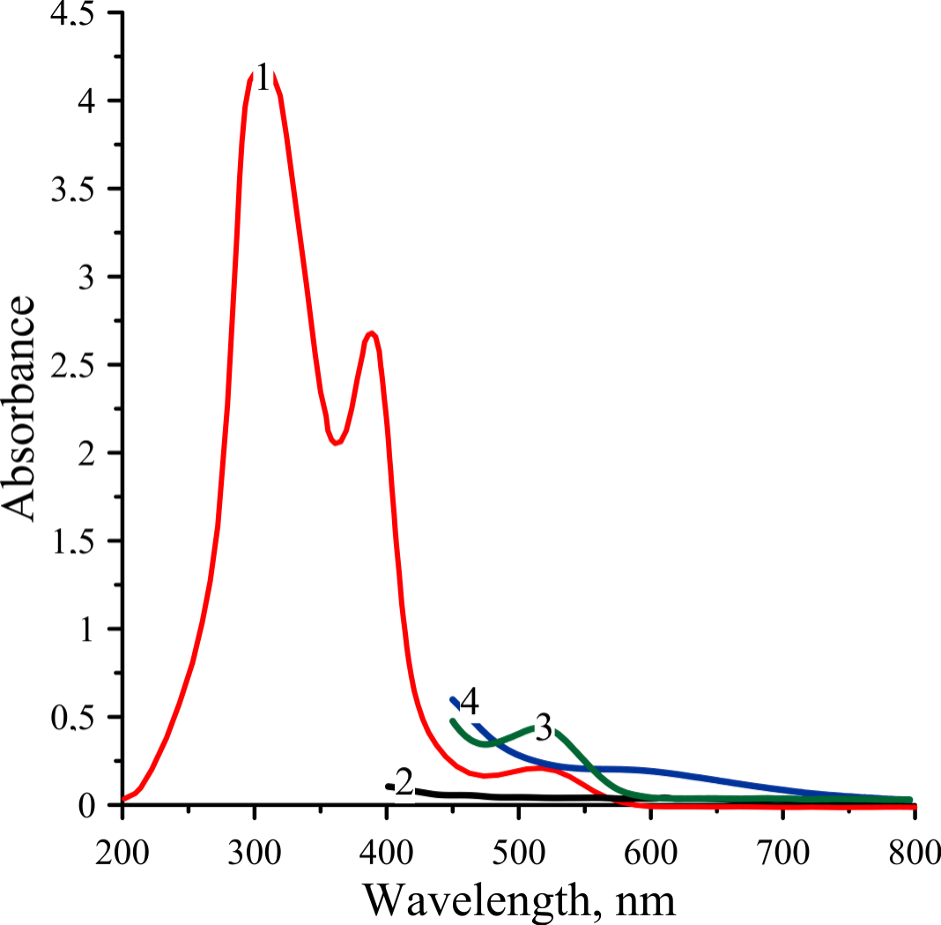
Absorption spectra in the UV and visible spectral region: 1) bis(cyclopentadienyl)titan dichloride (*n*-hexane, 0.4 mmol/L); 2) diethylaluminum chloride (*n*-hexane, 2.5 mmol/L); 3) Cp_2_TiCl_2_·AlEt_2_Cl (toluene, 10 mmol/L, Ti/Al ratios is 1:1, immediately after mixing); 4) Cp_2_TiCl_2_·AlEt_2_Cl (toluene, 10 mmol/L, Ti/Al ratios is 1:1, 10 minutes after mixing).

It is known that the catalytic activity of the Cp_2_TiCl_2_/organoaluminum compound is determined by the molar ratio of the components of the catalytic system [15]. The rate of transformation in the system depends both on the Al:Ti molar ratio and on the temperature [16]. UV spectra of toluene solutions of Cp_2_TiCl_2_ and AlEt_2_Cl (Figure 1) in the visible region at ambient temperature clearly demonstrate that during the first minute of the reaction an intermediate compound is formed, which gradually decomposes with formation of the blue complex [15–16].

The complexation between the organoaluminum compound and Cp_2_TiCl_2_ was further confirmed using ^1^H NMR spectroscopy [17–18].

**Figure 2:**
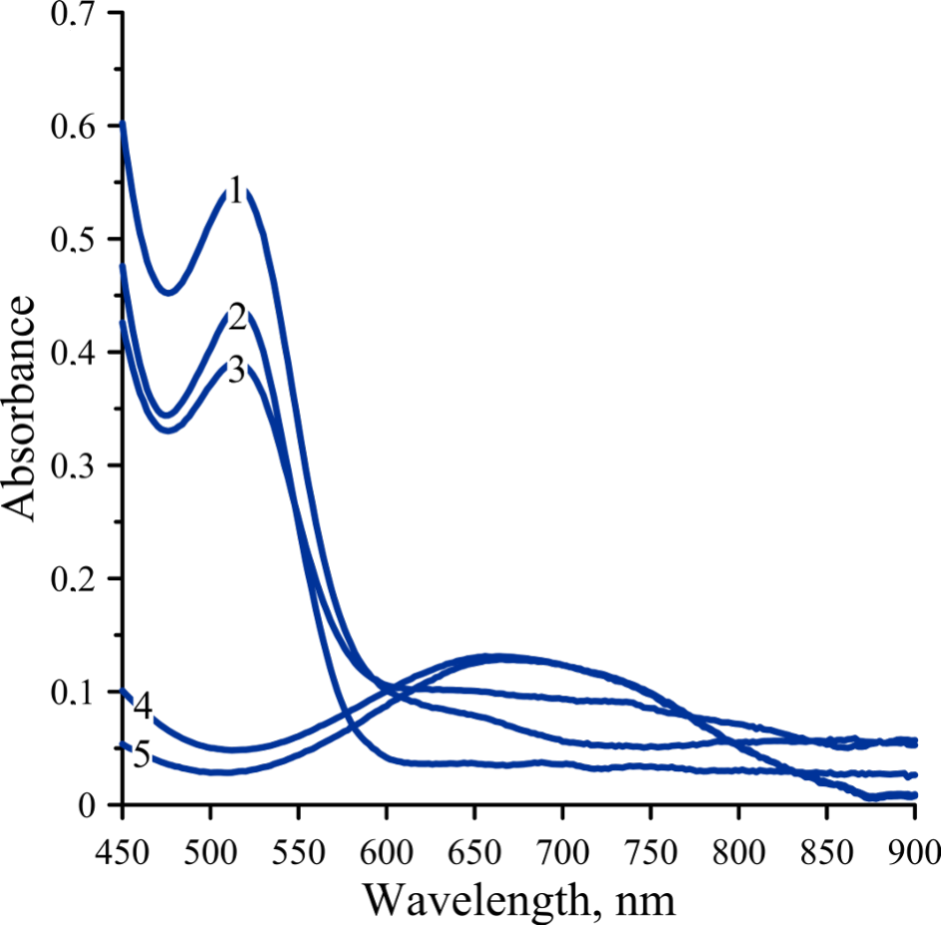
Absorption spectra in the visible spectral region: 1) Cp_2_TiCl_2_·AlEt_2_Cl (toluene, 10 mmol/L, Ti/Al ratios is 1:0.5); 2) Cp_2_TiCl_2_·AlEt_2_Cl (toluene, 10 mmol/L, Ti/Al ratios is 1:0.7); 3) Cp_2_TiCl_2_·AlEt_2_Cl (toluene, 10 mmol/L, Ti/Al ratios is 1:0.9); 4) Cp_2_TiCl_2_·AlEt_2_Cl (toluene, 10 mmol/L, Ti/Al ratios is 1:1); 5) Cp_2_TiCl_2_·AlEt_2_Cl (toluene, 10 mmol/L, Ti/Al ratios is 1:1.5). All spectra correspond to time 40 minutes after mixing.

The influence of the Ti/Al ratio was previously discussed [15,19]. Nonetheless, we studied the effect of the Ti/Al ratio on the formation of an absorption band at 700 nm (Figure 2). From the obtained data it follows that the absorption band at 700 nm appears only at Ti/Al ratios above 1:1, therefore, the ratio of Ti/Al equal to 1:1.5 was further used.

**Figure 3:**
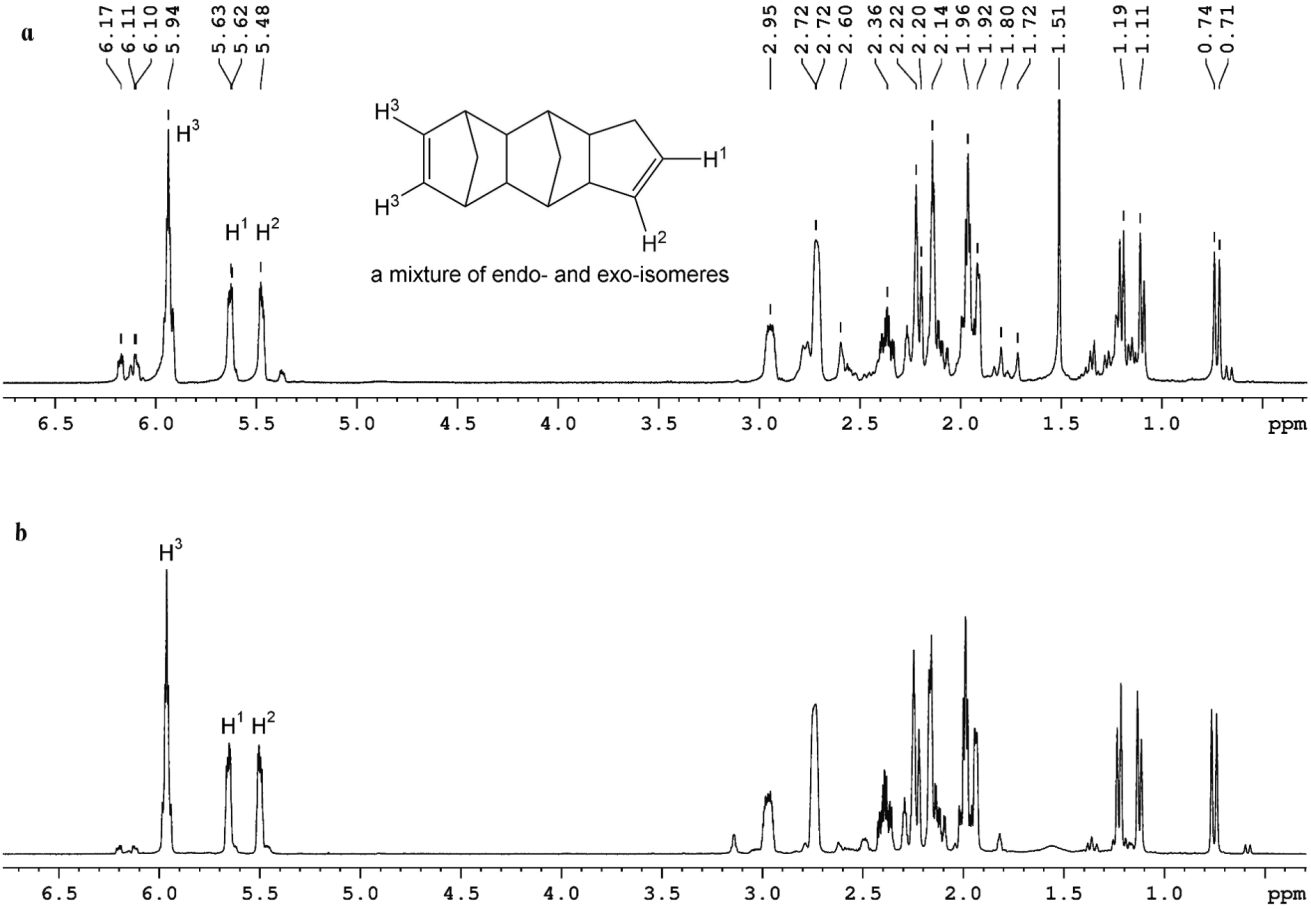
^1^Н NMR spectra of tricyclopentadiene (a) and the interaction product between Cp_2_TiCl_2_ and AlEt_2_Cl with dicyclopentadiene (b).

During this complex formation, generation of cyclopentadiene (CPD) trimers, resulting from the interaction between the cyclopentadiene ring of bis(cyclopentadienyl)titanium dichloride and dicyclopentadiene, occurs. Figure 3 presents the ^1^Н NMR spectra of the product formed in the reaction mass during the polymerization of DCPD in hexane (DCPD concentration of 1.5 mol/L, concentration of the catalyst system of 2.5 mmol/L, Ti/Al ratio is 1:1.5). After removing the polymer precipitate from solution, the remaining product is identified as a CPD trimer. The amount of trimer formed is small and amounts to 1–3% of the total DCPD taken per reaction. The appearance of interaction products of DCPD and the catalytic system generating the CPD trimer was unexpected. Typically, the CPD trimer is formed under more severe conditions, for example, at high temperatures ≈180 °C, (see Figure 3).

This was confirmed by NMR analyses of the interaction products between the complex of bis(cyclopentadienyl)titanium dichloride and diethylaluminum chloride with dicyclopentadiene (Figure 3b). The NMR spectrum of tricyclopentadiene obtained via condensation of dicyclopentadiene and cyclopentadiene is presented for comparison (Figure 3a).

**Scheme 1:**
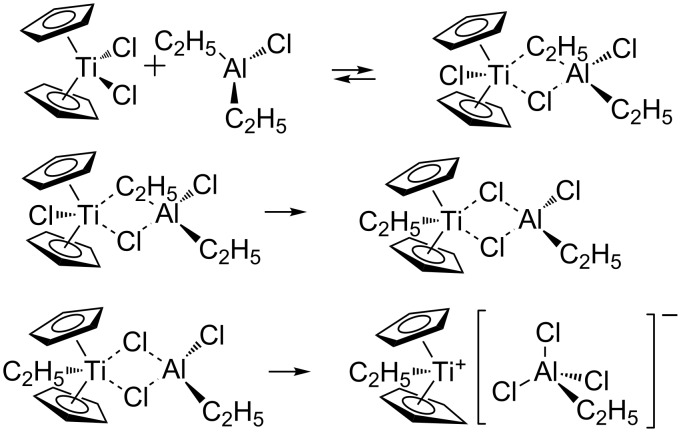
Mechanism of alkylation of Cp_2_TiCl_2_.

Dialkyl derivatives of aluminum very easily alkylate Cp_2_TiCl_2_. Alkylation can occur according to the following mechanism (Scheme 1):

During the interaction of the intermediate complex with cyclo- and dicyclopentadiene, generation of metal carbene species is possible, which can also take part in the formation of polydicyclopentadiene. Already in the work of Grubbs and others [20–23], the possibility was pointed out of the formation of simple structures with a carbene bond via interaction of organometallic transition metal complexes with organic aluminum compounds. The formation of such unstable bis(cyclopentadienyl)titanium dichloride complexes with a Ti=CHR fragment is possible as well in this case. The obtained complex is polarized in such a way that the metal has a positive charge, and the carbon atom has a negative charge [23]. It is assumed that after the formation of such complexes, they initiate the metathesis polymerization of dicyclopentadiene.

In the UV–vis spectrum of Cp_2_TiCl_2_, two maxima are observed at 388 and 516 nm. It is known that when a solution of AlEt_2_Cl is added to a Cp_2_TiCl_2_ solution, the maxima at 388 and 516 nm will disappear and a new band will appear in the region of 580 nm [15–16].

Mixing of toluene solutions of Cp_2_TiCl_2_ and AlEt_2_Cl demonstrates also a change in the visible region at ambient temperature and with the increase of the AlEt_2_Cl content the band at 516 nm, characteristic for Cp_2_TiCl_2_, disappears. As a result, a new band appears in the region of 570–610 nm, confirming the formation of an intermediate complex between Cp_2_TiCl_2_ and AlEt_2_Cl, however, this only occurs when an excessive amount of diethylaluminum chloride is present in solution.

Hence, the band with maximum absorption in the region of 580 nm is assigned to the intermediate complex Cp_2_TiCl_2_·AlEt_2_Cl, which is formed when solutions of Cp_2_TiCl_2_ and AlEt_2_Cl are mixed.

**Figure 4:**
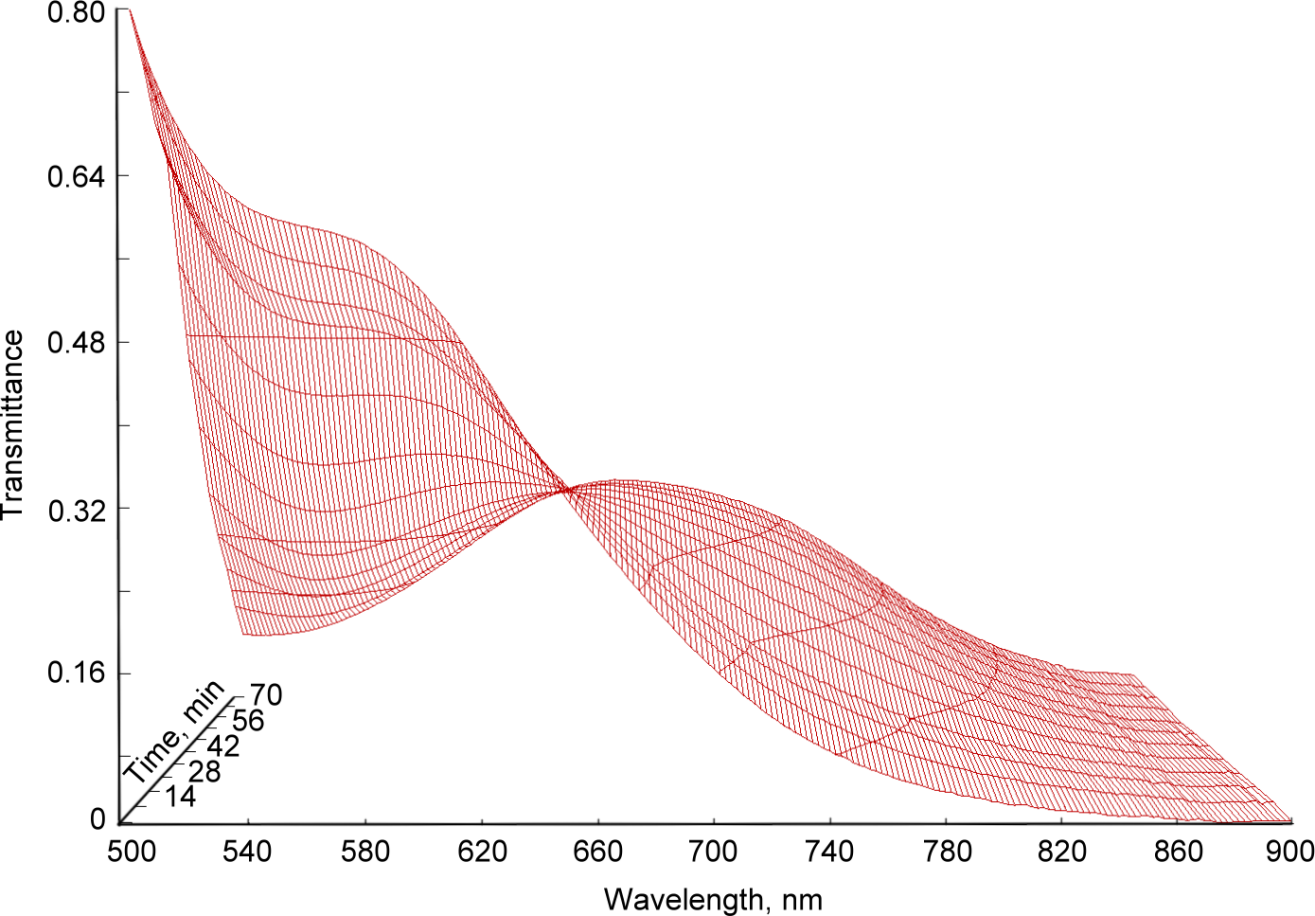
Visible spectra of a mixture of Cp_2_TiCl_2_ and AlEt_2_Cl as function of time.

The stability of the formed complex was investigated using visible spectroscopy and the obtained spectra are depicted in Figure 4.

A clear change as a function of time can be observed by the decrease of the band at 580 nm. Moreover, a shift of the absorption band towards 700 nm and a broadening can be observed. The final visible spectrum (Figure 2, curve 5) corresponds to [Cp_2_TiEt]^+^·[AlEtCl_3_]^−^, the blue complex. Indeed, as reported in previously published papers [15–16], the colored blue complex under these conditions is caused by a compound containing Ti(III) or Ti(IV). This compound corresponds to the final [Cp_2_TiEt]^+^·[AlEtCl_3_]^−^ complex.

The presence of an isosbestic point at 656 nm indicates the presence of only two absorbing complexes, which transfers one into the other.

### Polymerization of DCPD applying the complex based on Cp_2_TiCl_2_

Polymerization of DCPD, applying the homogeneous catalytic system consisting of Cp_2_TiCl_2_ and AlEt_2_Cl, was performed by adding a fresh solution of the catalytic system to a toluene solution of the monomer. However, before adding the catalytic complex, the monomer solution was placed in an adiabatic mixing reactor until the temperature was stabilized. To limit the development of the polymer chain and as a deactivator of the catalyst system, propylene oxide was used. The polymerization of DCPD was carried out under the following conditions: ratio of Ti/Al 1:1.5, concentration of the complex Cp_2_TiCl_2_/AlEt_2_Cl from 2 to 10 mmol/L, and concentration of DCPD 1.5 mol/L.

**Figure 5:**
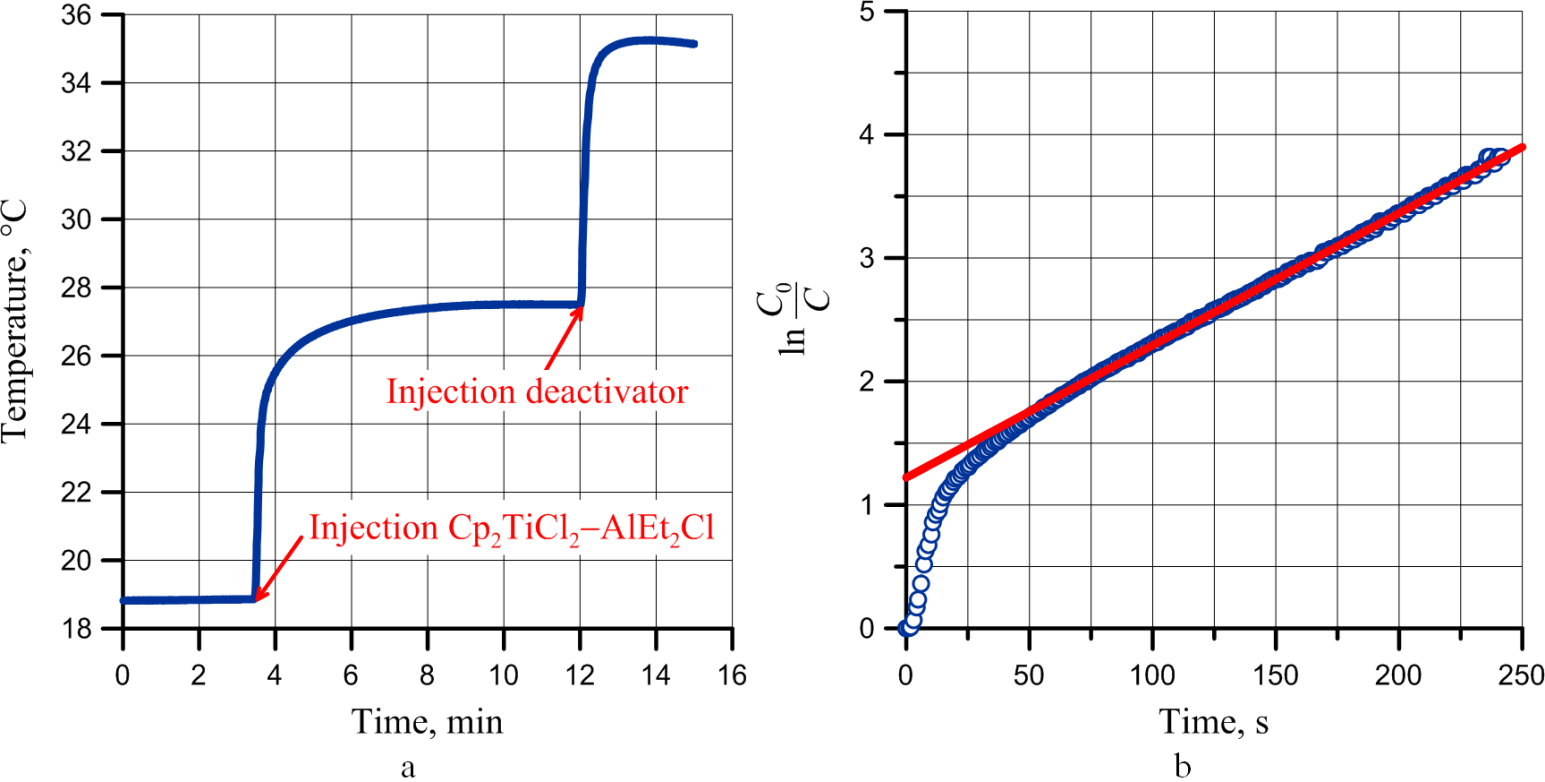
Thermometric curve of DCPD polymerization using the catalyst system based on Cp_2_TiCl_2_ (a) and its semi-logarithmic plot of ln *C*_0_/*C* vs time (b).

Figure 5 shows a typical thermometric curve for the polymerization of DCPD (Ti/Al ratio 1:1.5, concentration of Cp_2_TiCl_2_/AlEt_2_Cl complex 10 mmol/L, concentration of DCPD 1.5 mol/L). Based on the assumption that the stage of chain growth proceeds as a pseudo-first order reaction, for every experiment, we calculated the observed reaction constant using the experimental curve in semi-logarithmic coordinates (Figure 5b) [24]. The value of the observed constant of DCPD polymerization rate in the toluene solution applying the catalyst system amounts to 0.011 mol^−1^∙s^−1^.

**Scheme 2:**
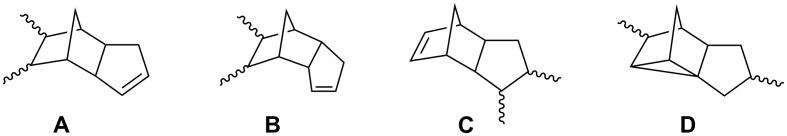
The structures formed as a result of the cationic polymerization of dicyclopentadiene.

Furthermore, it is assumed that in this case, cationic polymerization of DCPD proceeds via one of the double bonds. With the participation of the double bond from the norbornene ring of dicyclopentadiene in the double bond reaction, as a result of the rearrangement of the active site, structures of both *exo-* and *endo-*polydicyclopentadiene (**A** and **B**, see Scheme 2) can be formed [1,10].

At the same time, with participation in the reaction of the cyclopentene double bond, one of the options may be the formation of the **D** units (Scheme 2) as a result of the transannular rearrangement of the growing carbocation [1]. As it was found, **A**-type units (up to 70%) dominate in the structure of polymers formed as a result of cationic polymerization. The number of formed **B**- and **C**-type units is about the same.

**Scheme 3:**
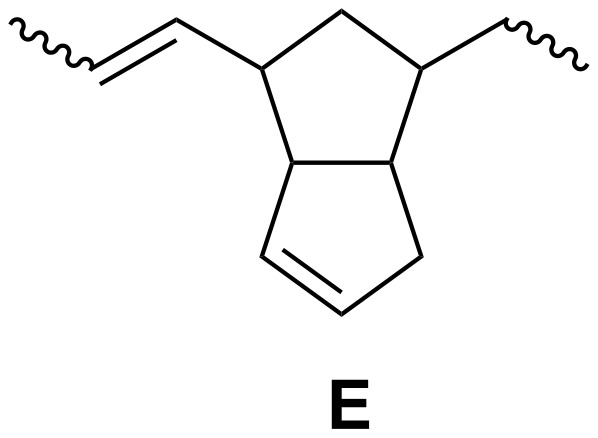
The units resulting from ROMP of dicyclopentadiene.

**Scheme 4:**
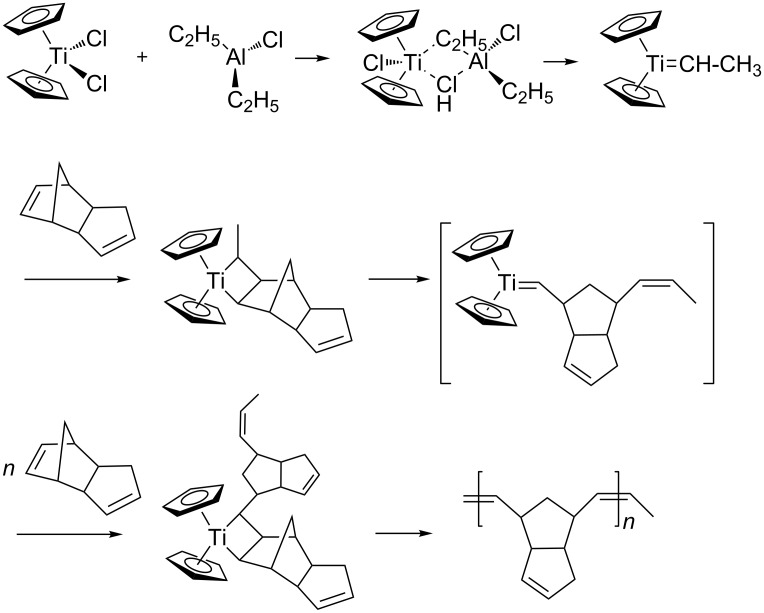
Mechanism of ROMP dicyclopentadiene.

In addition, a small amount of polymer **E** units (5–7%) is also formed as a result of the metathesis polymerization of dicyclopentadiene (see Scheme 3). It was reported [20,22–23,25–26] that the Tebbe reagent, as shown, is a precursor of titanium carbene, which reacts with R-olefin and a Lewis base to form stable crystalline titanacyclobutanes. Both titanium carbene and titanacycles are ROMP catalysts (Scheme 4).

PDCPD polymers were obtained by precipitation in ethanol, dried and characterized by FTIR, NMR, and GPC.

**Figure 6:**
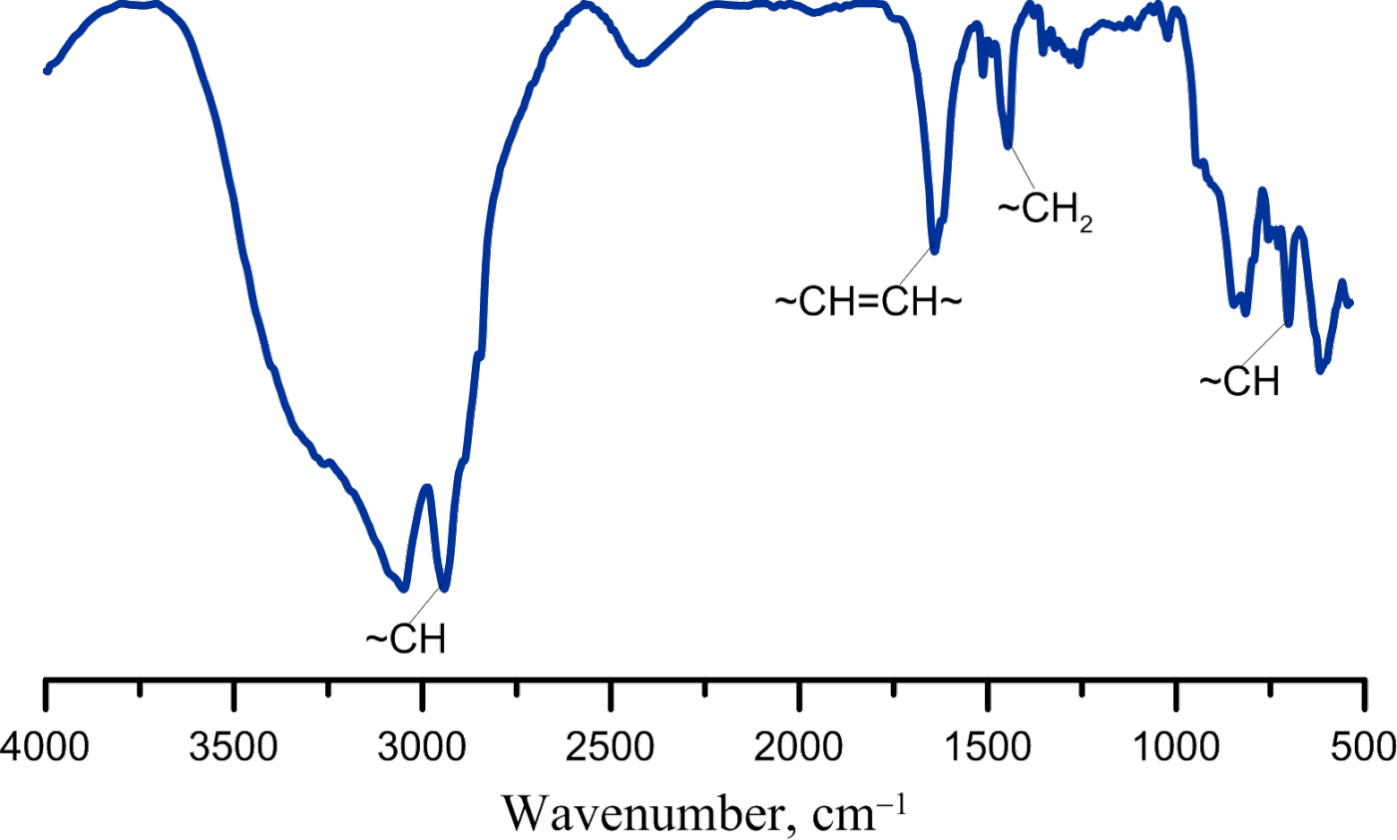
FTIR spectrum of PDCPD obtained in toluene with the catalyst system based on Cp_2_TiCl_2_ and AlEt_2_Cl.

Figure 6 displays a typical infrared spectrum of PDCPD obtained with the catalyst system based on Cp_2_TiCl_2_ and AlEt_2_Cl.

This spectrum displays specific regions, e.g., the regions from 690 to 800 cm^−1^ can be assigned to out-of-plane deformation vibrations of the C–H group. The band at 1440 cm^−1^ points out the presence of CH_2_ groups. The bands in the region of 1620 cm^−1^ confirm the presence of C=C groups, while the absorption band at 2990 cm^−1^ demonstrates the presence of CH–CH_2_ groups in the ring.

**Figure 7:**
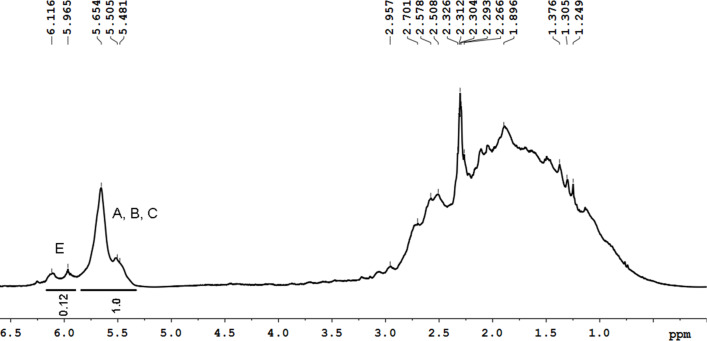
^1^Н NMR spectrum of PDCPD obtained with the catalytic system based on Cp_2_TiCl_2_ and AlEt_2_Cl.

Figure 7 shows the ^1^H NMR spectrum of the obtained polymer, in which the region from 0.5 to 3.5 ppm is assigned to aliphatic protons. This region contains a wide signal corresponding to the superposition of resonances of –СН and –СН_2_ groups of cyclopentene and cyclopentane rings. The region from 5.0 to 6.3 ppm contains several wide signals corresponding to resonances of protons of double bonds of the polymer chain and the cyclopentene ring (see Scheme 2 and Scheme 3).

According to GPC, the molecular weight of the polymers was in the range of (10–50)·10^3^ with a molecular weight distribution of about 2–3.

**Figure 8:**
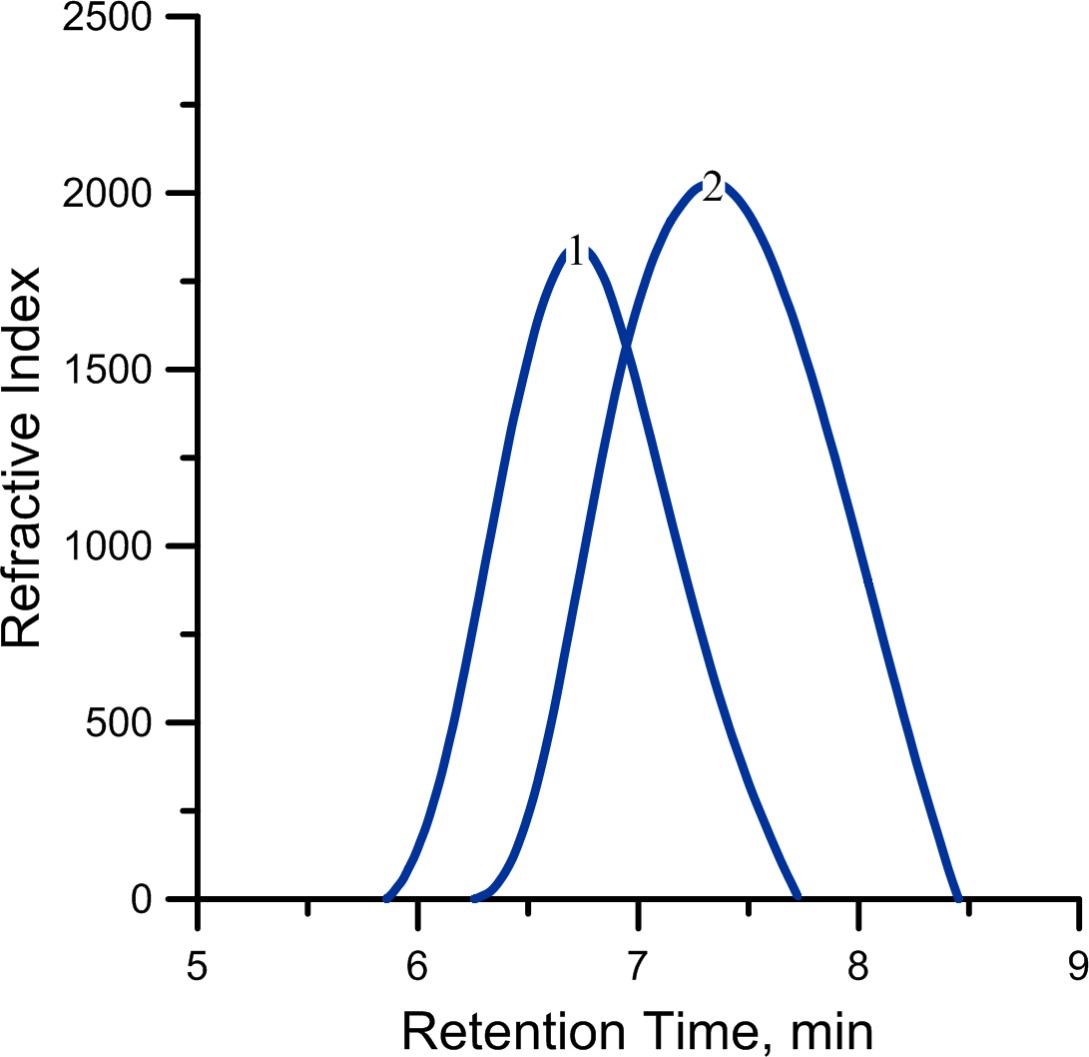
GPC traces for two samples of DCPD polymers obtained at a concentration of Cp_2_TiCl_2_/AlEt_2_Cl complex 2 mmol/L (curve 1) and 10 mmol/L (curve 2).

Figure 8 displays the GPC traces for two samples of DCPD polymers obtained at a concentration of Cp_2_TiCl_2_/AlEt_2_Cl complex 2 mmol/L (curve 1) and 10 mmol/L (curve 2). The remaining conditions are the same: Ti/Al ratio 1:1.5, concentration of DCPD 1.5 mol/L. *M*_w_(1) = 5.13·10^4^, *M*_n_(1) = 2.69·10^4^, PDI(1) = 1.91; *M*_w_(2) = 1.32·10^4^, *M*_n_(2) = 4.84·10^3^, PDI(2) = 2.73 of additional monomer.

### Oxidizing of thin layers of PDCPD in air

**Figure 9:**
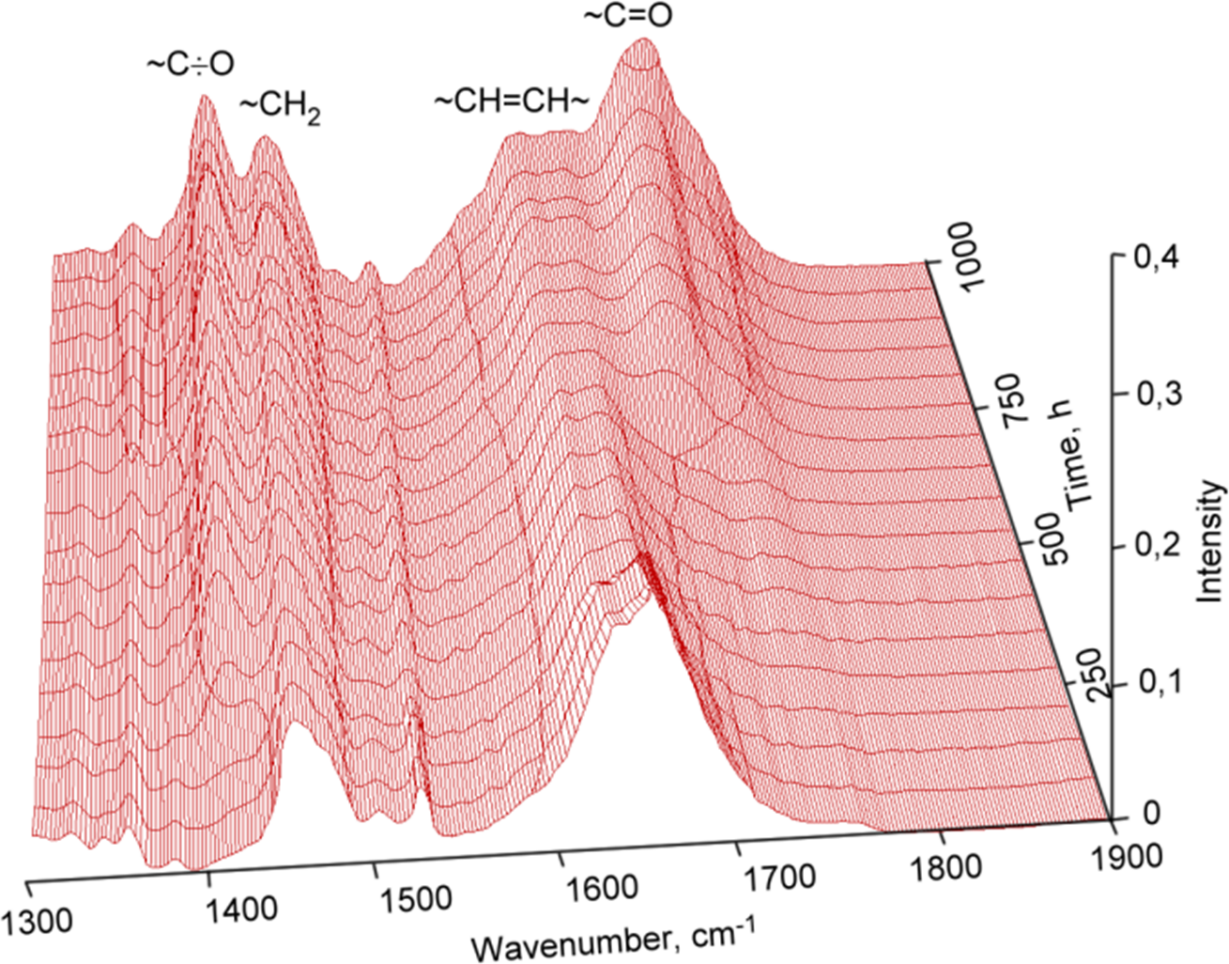
IR spectra of cationic polymerized dicyclopentadiene taken after certain periods of time exposed to air.

Oxidation in air of olefinic bonds in a thin layer of polydicyclopentadiene is a gradual process and can be observed by the increase of intensity of the vibration band of carbonyl and hydroxy groups in the infrared spectra of the polymers (Figure 9). The wide band at 3400 cm^−1^ belongs to stretch vibrations of hydroxy groups located at various carbon atoms in the main polymer chain. The intensity of the deformation vibration of the carbonyl groups also increases at 1700 cm^−1^, while the intensity of the deformation vibration of the double bonds decreases at 1620 cm^−1^.

Figure 9 reveals that structural changes gradually happen during the exposure time of polydicyclopentadiene thin layers in the air as a result of the oxidation of double bonds. A new vibrational band at 1410 cm^−1^ in the IR spectrum appears which is originating from the primary radicals which are formed alongside the chain initiation.

**Figure 10:**
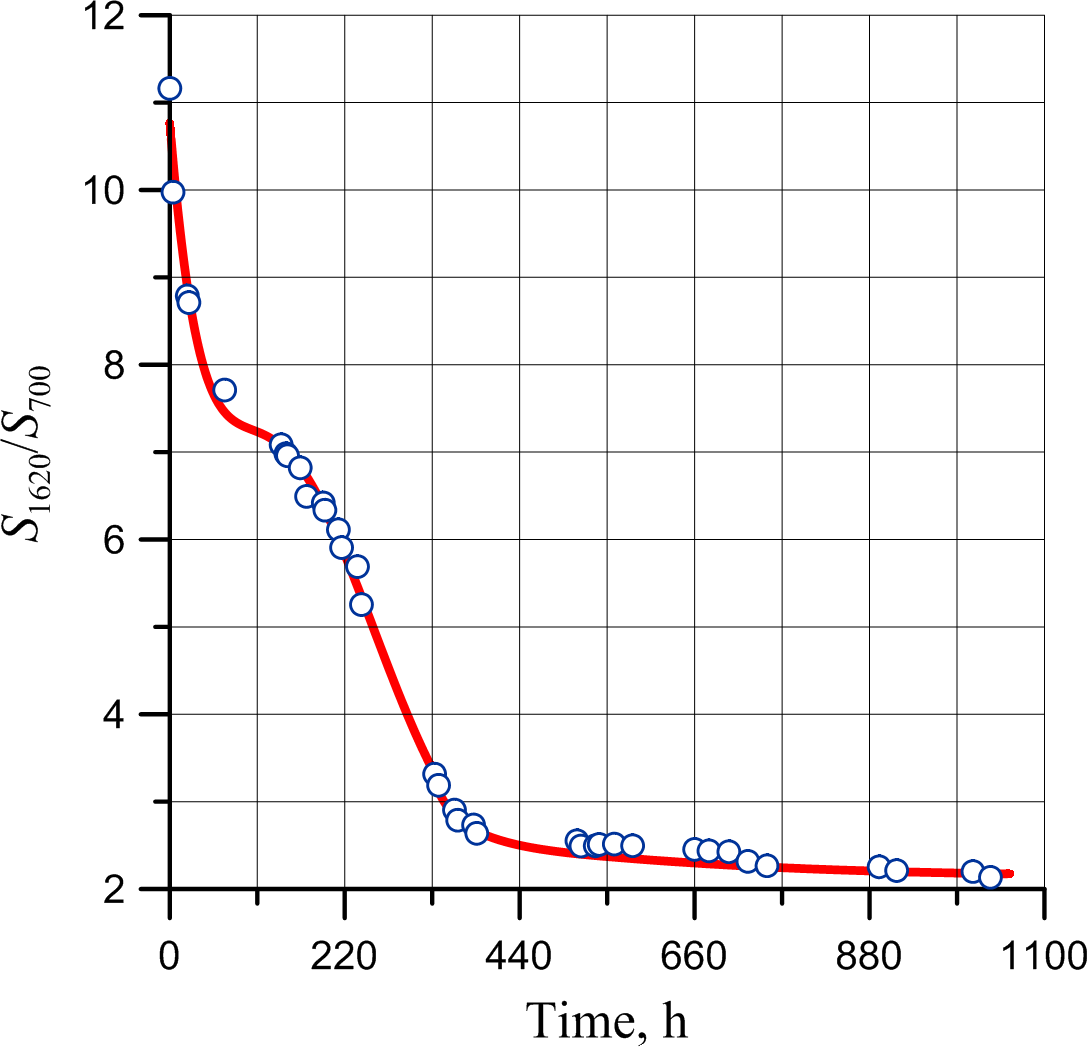
Correlation of intensities of vibrational bands at 1620 and 700 cm^−1^ and layer exposure time in air at ambient temperature.

The kinetics for the oxidation in air at ambient temperature of PDCPD layers was studied applying the changes in intensity of the double bond deformation vibrations. Figure 10 shows the kinetic curve of the PDCPD oxidation obtained from the correlation between the changes of the relative intensity of double bond deformation vibrations and the layer exposure time in air at ambient temperature.

The correlation presented in Figure 10 demonstrates that the kinetics of double bond consumption during oxidation occurs in two stages. During the first stage, the chain (formation of primary radicals) initiates, and then the chain process of PDCPD oxidation follows.

Various mechanisms of chain initiation are possible, e.g., the formation of primary free radicals initiating the chain reaction of polymer oxidation (Equation 1). More often, the chain initiation step is described as a bimolecular interaction between oxygen and a monomer unit of the polymer:

[1]
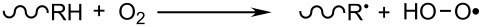


**Figure 11:**
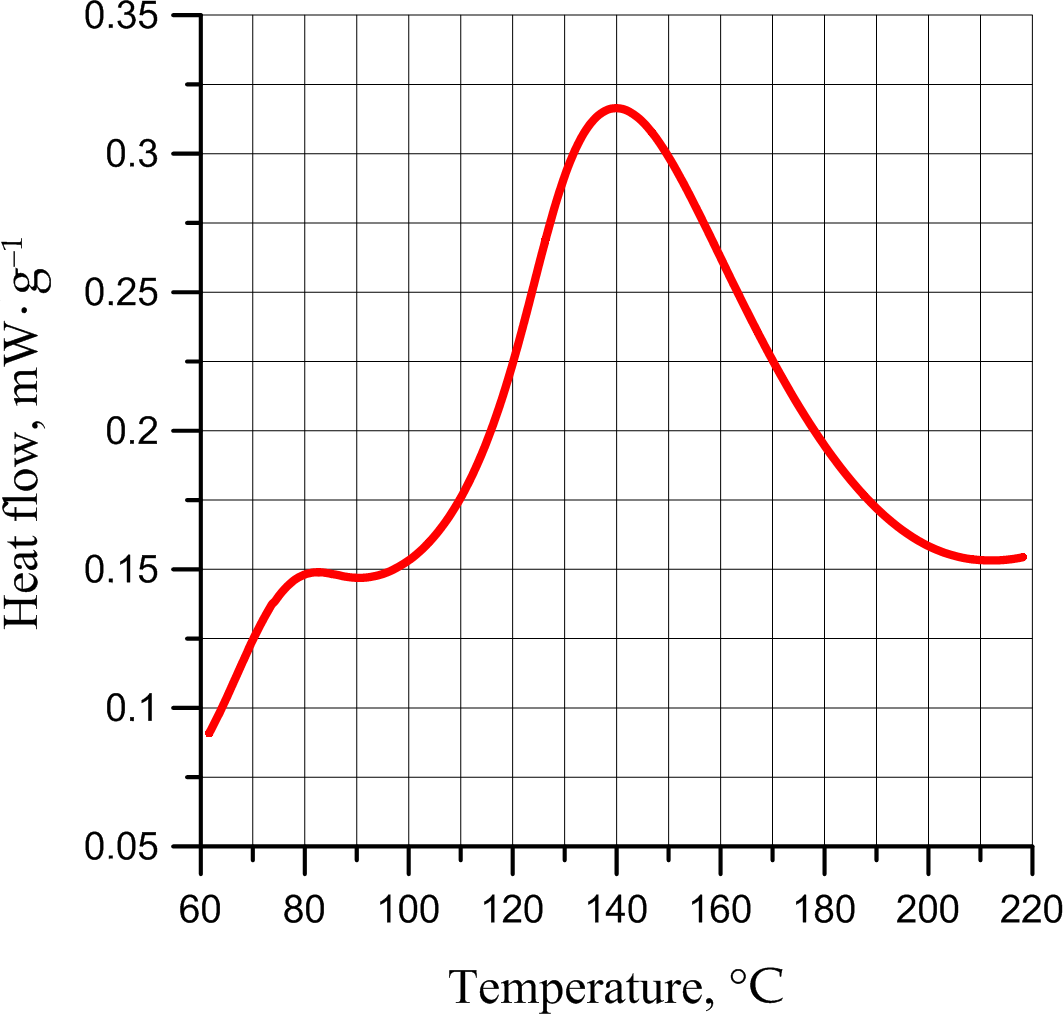
DSC exotherm for PDCPD subjected to air oxidation for 700 hours.

Accumulation of peroxides in the polymer layer is confirmed by DSC analysis of films subjected to air oxidation for 700 hours (Figure 11).

From the DSC curve (Figure 11), at 140 °C an exothermic peak can be observed corresponding to the decomposition of peroxides accumulated during the oxidation of PDCPD. The peak value of heat flux is slightly lower than that given in [27], which is explained by the slower diffusion of oxygen into the polymer film from air and the lower temperatures of the oxidation of thin PDCPD films in this study.

**Figure 12:**
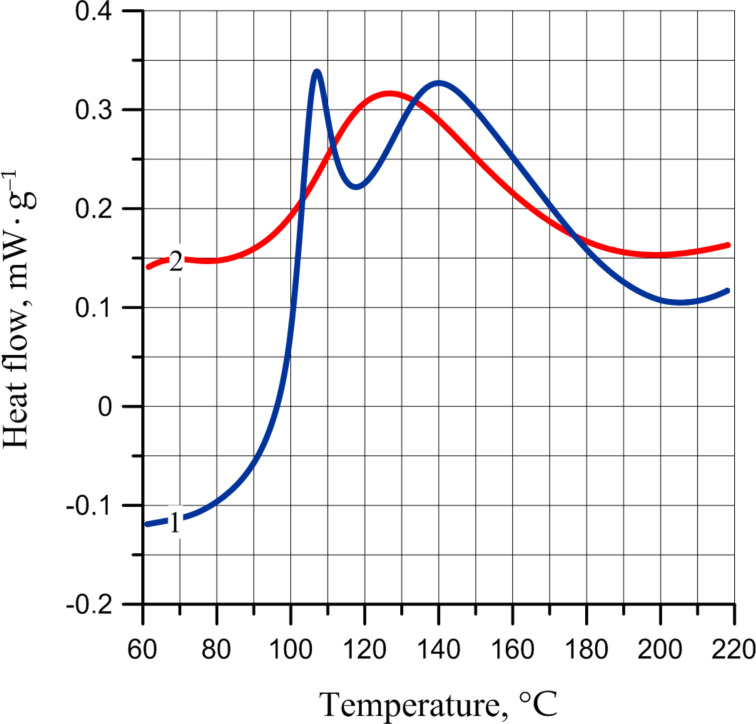
DSC exotherm for PDCPD subjected to unexposed film: 1) in air atmosphere; 2) in argon.

In our opinion, the peak at 80 °C can correspond to the processes of oxidation of -C=C- bonds in the polymer chain due to adsorbed oxygen. In the DSC of unexposed film, this peak is absent. However, the DSC of unexposed film in air atmosphere (Figure 12) shows that the oxidation and decomposition of peroxides formed during the oxidation of polydicyclopentadiene occur simultaneously.

**Scheme 5:**
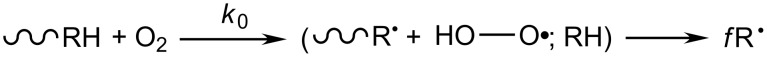
Possible radical formation in the reaction (1).

НО–O• radicals formed during this process can react with monomer components near them, thus, forming R• radicals and recombine with primary R• radicals. Therefore, the theoretical yield of radical formation in the reaction (1) ranges between 0 and 2, and can be conveniently described as the reaction given in Scheme 5.

Impurities remaining in the polymer after its purification can participate in the initiation of the chain oxidation. These impurities can include initiator or catalyst residues, metal impurities with mixed valences, in particular, those of iron and copper, peroxy and carbonyl group-containing compounds.

**Scheme 6:**
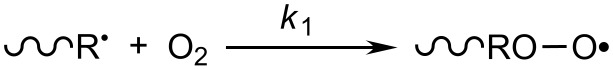
The first step of the chain propagation.

Unlike the initiation, the steps of chain propagation during polymers oxidation are well studied [28]. The first step of chain propagation consists of the interaction of the free R• radical with oxygen (Scheme 6) and occurs at an observable rate at low temperatures.

In a kinetic mode, the polymer oxidation rate is limited by the kinetic steps of the chain process, indicating that oxygen is quickly transferred from the gaseous phase into a polymer (macro-diffusion) and does not limit the process rate. Otherwise, when oxygen is slowly supplied into the sample, the process rate is limited by the diffusion, and the oxidation takes place in a diffusion mode. The reaction kinetics is consecutive and hence, it is characterized by a wide range of rate constants and can be described by the following equation:

[2] $$\frac{dc}{dt}=D\left(\frac{\partial^{2}c}{\partial x^{2}}\right)-kc$$

where the first element on the right defines the oxygen diffusion rate into that element, and the second element defines the rate of its chemical reaction.

The univocal criterion of the diffusion mode is the correlation of the oxidation rate and the sample size (layer thickness, ball or cylinder diameter, etc.). If the sample is plate-shaped and 2*l* thick and its linear size is much bigger than 2*l*, then the concentration of oxygen in each element of the sample at time *t* is determined by following Equation 2.

However, under stationary conditions, when the oxygen supply rate into the sample during diffusion equals its consumption rate in the chemical reaction, then the oxygen concentration in each element is independent of the time, i.e.,


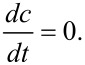


Hence, Equation 2 can be reorganized as:


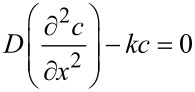


Under boundary conditions (*с* = *с*_0_ as *х* = 0 and *dc/dx* = 0 as *х* = *l*), the solution of this equation gives the oxidation rate as a ratio to a polymer mass unit [28]:


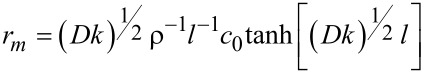


where *D* is the oxygen diffusion coefficient; ρ is the polymer density and for *l* → ∞ (*r_m_*)_∞_ → 0, while *l* = 0 *r_m_* = *kc*_0_, i.e., oxidation transfers into a kinetic mode. In this case, the value of *k* is 1.6·10^−3^ h^−1^.

**Figure 13:**
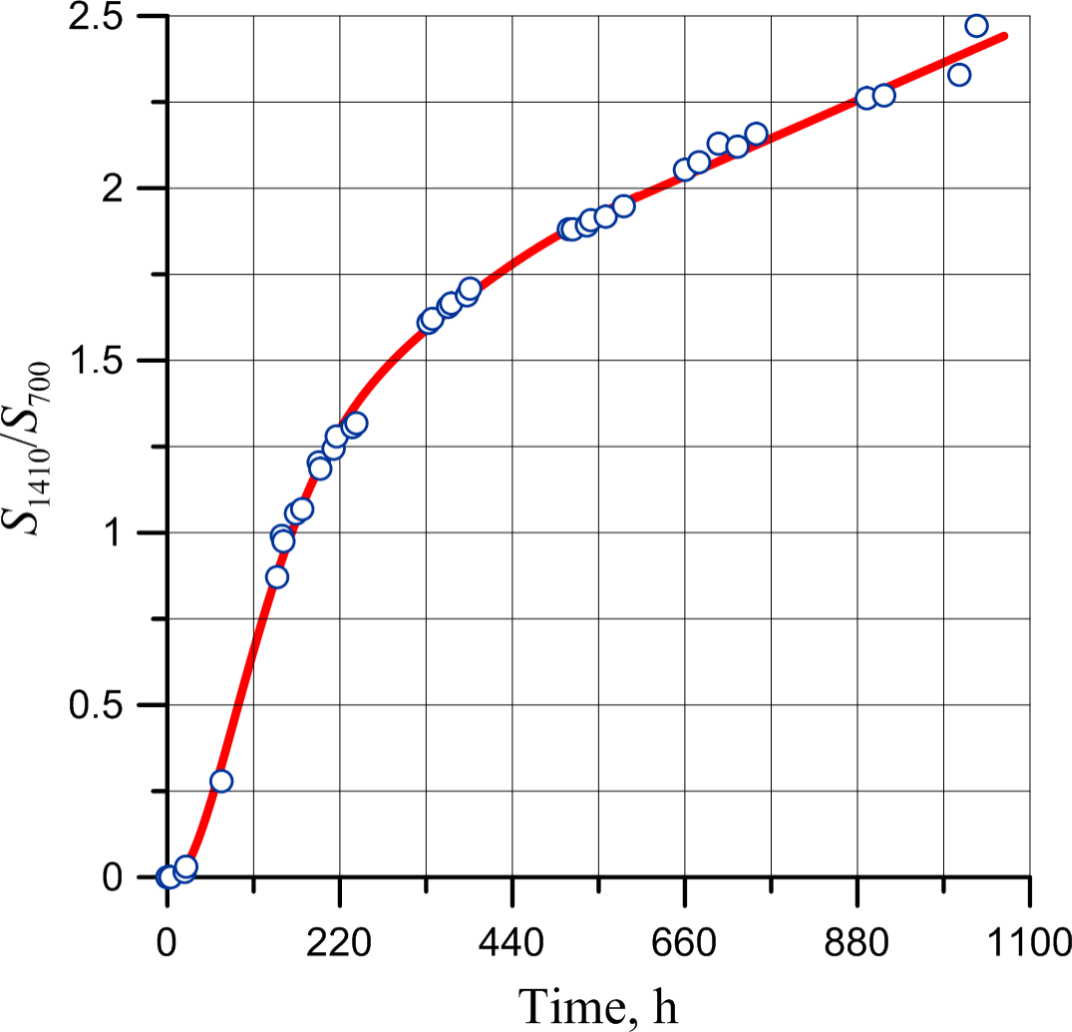
Dependence of intensities of adsorption bands at 1410 and 700 cm^−1^ and dwell time of the layer in air at ambient temperature.

Equation 2 helps to understand the appearance of the curves of the dwell time of a layer in air at ambient temperature (Figure 13).

According to the classical theory of oxidation of polymers, the formation of primary radicals occurs predominantly, and only when they are formed, further oxidation of the -C=C- bonds occurs with the aid of the peroxide radicals formed. However, crosslinking of polymer chains occurs along with oxidation processes, which leads to compaction of the polymer structure and reduction of the mobility of the polymer chains. This adversely affects the rate of penetration of air oxygen through the layer of the structured polymer. As a result, physical adsorption of oxygen and its transport through the polymer layer becomes the slowest process, which leads to a change in shape of the kinetic curve of the accumulation of peroxide radicals (Figure 13).

**Figure 14:**
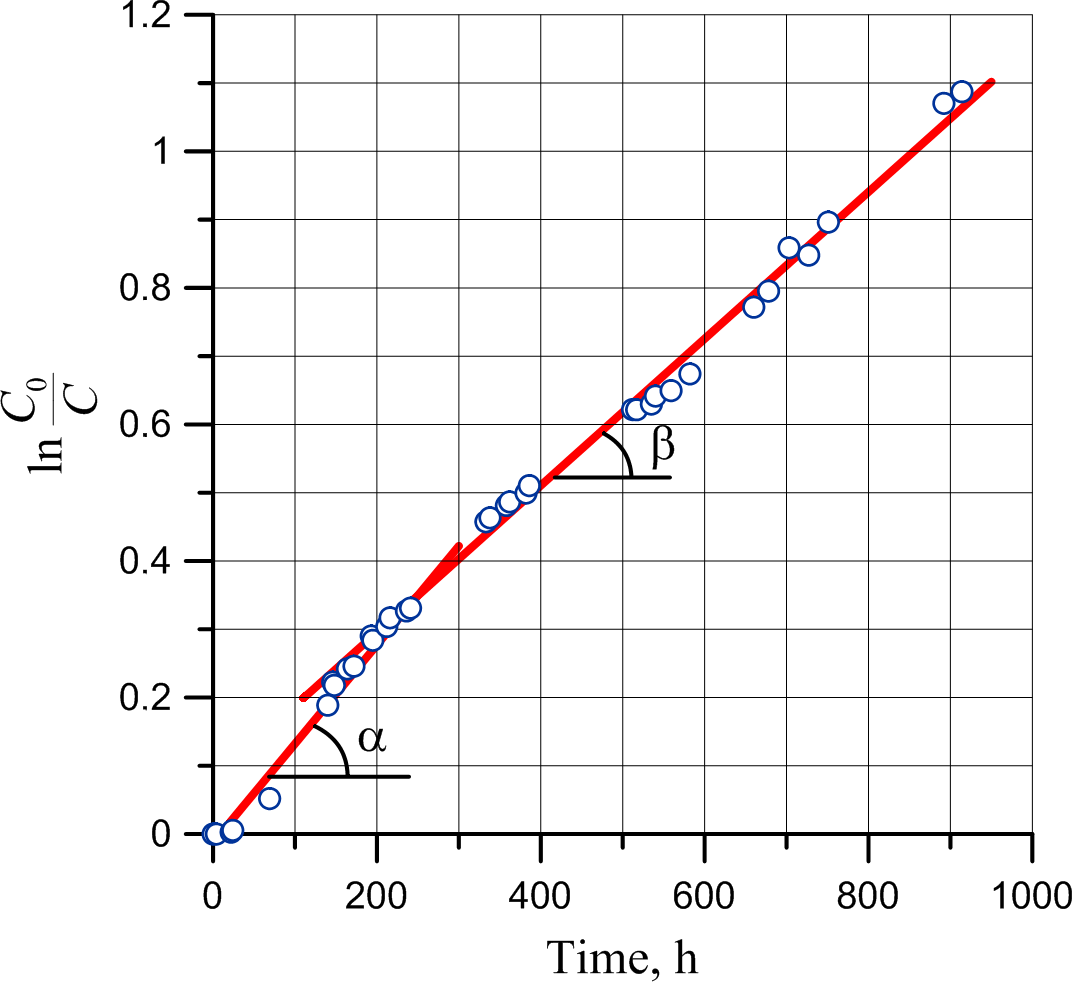
Semi-logarithmic kinetic curve of PDCPD oxidation in air (thin layer on silicon) with respect to intensities of adsorption bands at 1410 and 700 cm^−1^.

The curve in Figure 13 averages the experimental points of the oxidation process and is a result of two interpolations – a curve in the initial part (up to about 500 hours) and a straight line for the rest of the time interval. In fact, the transition to the diffusion mode occurs much earlier, as can be clearly seen from the semi-logarithmic curve (Figure 14).

A number of PDDCP studies [29] indicate the possibility of the formation of a thin film of a chemically modified polymer, which reduces its permeability to corrosive media. We assume that in case of PDCPD oxidation, the formation of chemically modified polymer layers also occurs, which reduce the permeability of the film to oxygen.

The double bonds located on the surface of the polymer are capable of various addition reactions (bromination, epoxidation, oxidation) forming films of several tens or hundreds of nanometers thick on the surface. However, no further penetration of reactants into the deeper polydicyclopentadiene layers occurs [28]. It is this effect that causes the great chemical inertness of PDCPD in relation to aggressive media. Actually, since the initial part of the curve is exponential, then along with the increase of the duration of the layer oxidation, and while structuring is in progress, the process gradually transfers into the diffusion mode.

The transfer into the diffusion mode of the oxidation is shown by a semi-logarithmic curve when its slope changes (Figure 14).

**Figure 15:**
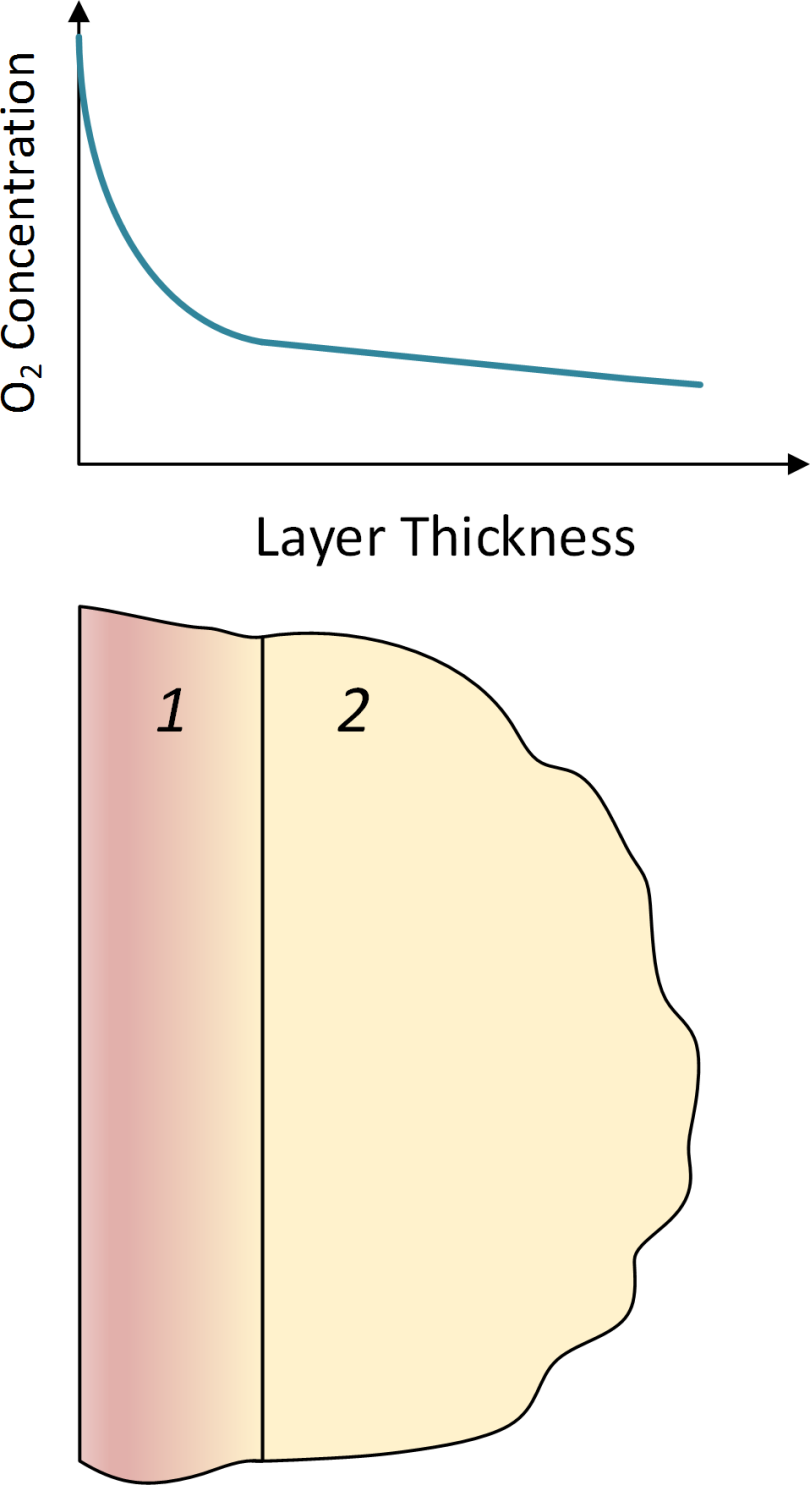
The distribution of oxygen concentration in the polymer layer: 1 – a layer of oxidized cross-linked polymer; 2 – a layer of non-oxidized polymer.

The oxygen concentration is maximal before the polymer layer; therefore, at a small depth of the layer, the rate of oxygen consumption is determined by the proceeding polymer oxidation reactions. However, the resulting film of oxidized crosslinked polydicyclopentadiene prevents further penetration of oxygen into the depth of the polymer layer (Figure 15).

At this stage in general, the oxidation process is limited by the diffusion of oxygen in the thickness of the polymer layer. The rate of oxygen consumption at the initial time point is influenced by many factors, of which the main factors are the formation and growth of the thickness of the oxidized cross-linked polymer layer on the film surface and the change in the rate of oxygen diffusion through the layer due to the changing properties of the polymer film. Later on, when the layer of oxidized cross-linked polymer is formed, the speed of the PDCPD oxidation process is limited only by the rate at which oxygen enters the polymer layer.

**Figure 16:**
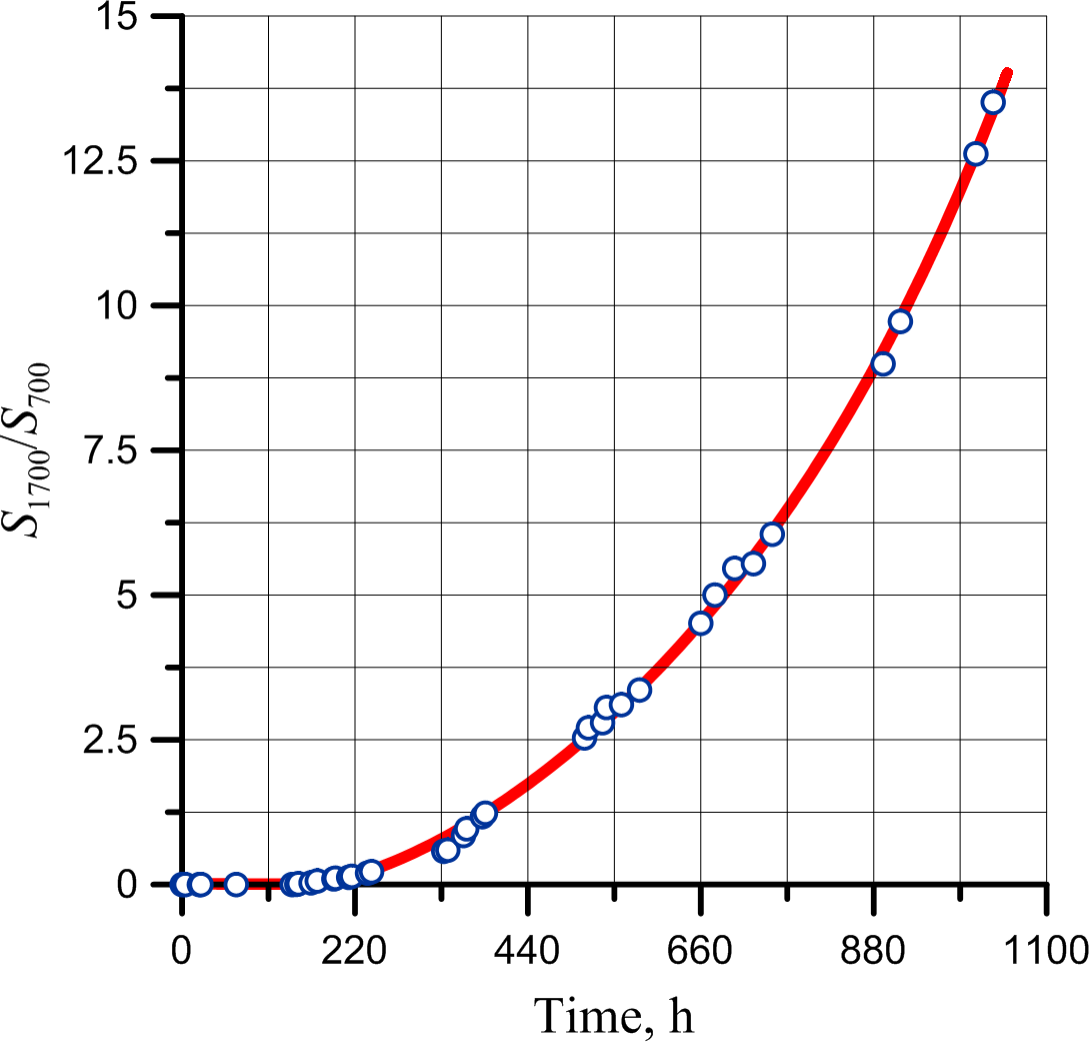
Dependence of the ratio of adsorption bands at 1700 and 700 cm^−1^ on the exposure time of the layer in air at ambient temperature.

At the same time, the accumulation of carbonyl and hydroxy group vibrations in the polymer does not occur immediately when the induction period is finished (Figure 16).

It is worth to mention that its induction period coincides with the passing of the first stage of double bond consumption in the polymer (Figure 10).

**Figure 17:**
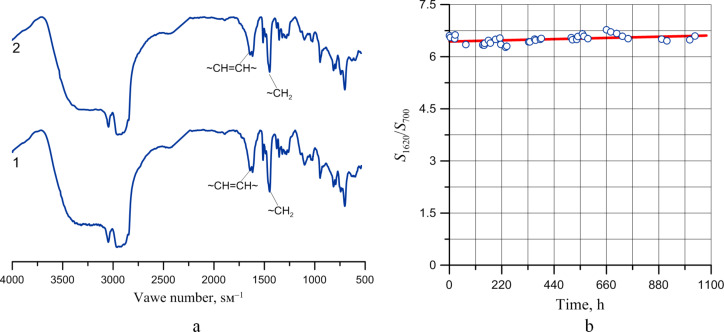
Infrared spectra (a) of products of cationic polymerization of DCPD, stabilized with an antioxidant, after 24 hours (curve 1) and 1030 hours (curve 2) after synthesis (thin layer on silicon wafer) and (b) the correlation of intensity ratios of adsorption bands at 1620 and 700 cm^−1^ vs layer exposure time in air at ambient temperature.

Finally, the abovementioned structural changes did not occur in the polymer which was stabilized by adding an antioxidant (Agidole-1 in the amount of 0.2% by mass). The infrared spectrum of the thin layer of the stabilized polymer (Figure 17a) does not change and no consumption of double bonds in the polymer can be detected (Figure 17b).

## Conclusion

This study reports regularities of DCPD polymerization in a toluene solution applying a catalytic system consisting of Cp_2_TiCl_2_ and AlEt_2_Cl. It was demonstrated that the use of an excessive amount of organoaluminum leads to the formation of stable charged blue complexes which initiate the cationic polymerization of dicyclopentadiene.

Polymer thin-film coatings of PDCPD obtained via cationic polymerization in air undergo oxidation and transformation. Oxidation in air of unsaturated bonds in layers occurs gradually and takes place during several weeks and comes amid with the growth of carbonyl and hydroxy group vibration bands in the infrared spectra. At the same time, structuring and isomerization occur in layers generating changes in their physical properties, in particular, the decrease of layer permeability for atmospheric air. In its turn, this leads to the transition of the oxidation from a kinetic mode into a diffusion one.

These structural changes do not occur in a polymer stabilized by adding an antioxidant in the studied period of time.

## Experimental

Dehydrated toluene, prepared according to a well-known procedure, was used as a solvent [30]. Polymerization of DCPD in toluene was carried out in a 100 mL adiabatic mixing reactor [31]. A thermometric method was used to study the kinetics of the process, which was carried out in adiabatic conditions with minor temperature change; hence, the thermometric curve is at the same time a kinetic plot [24]. The temperature was registered during the process with a digital thermometer, consisting of a platinum thin film resistance thermometer placed on a ceramic substrate and placed in a stainless steel thin-wall case.

The catalyst for cationic DCPD polymerization is a complex that is formed during the interaction of Cp_2_TiCl_2_ with AlEt_2_Cl. The estimated amount of Cp_2_TiCl_2_ (Sigma-Aldrich, 99% pure) was dissolved in toluene. AlEt_2_Cl was used as a solution in toluene with a concentration of 0.232 g/mL. All working solutions were obtained by diluting the stock solutions with dry solvent until the required concentration was obtained.

DCPD (Hangzhou Uniwise International Co., Ltd., 99% pure) was purified from stabilizers by distillation under reduced pressure (≈6,6 kPa).

All operations with monomer and catalyst were carried out in a glove box MBraun Labstar provided with an argon atmosphere.

UV–vis spectra of catalyst system solutions were registered by a spectrophotometer Thermo Scientific Evolution 201 using a wavelength range from 200 to 900 nm.

Infrared spectra of the polymer were registered applying an FTIR spectrometer Simeks FT-801 in the range from 500 to 4000 cm^−1^. A silicon plate with a diameter of 8 mm was applied to support the polymer film and degreased before use. Polymer films were applied by irrigation from 2–5% solutions of PDCPD in toluene, followed by drying at 25 °C under a nitrogen atmosphere (Binder VDL 23 Vacuum Drying Oven), with a gradual decrease in pressure at the end of the drying process.

The thickness of the polymer film was controlled so that the maximum light absorption in the wavelength range of 500–4000 cm^−1^ did not exceed 1.2 units of absorption (EP) and remained in the preferred range of 0.3–1.0 EP. The optimum thickness of the film of polydicyclopentadiene was 10 μm.

^1^H NMR spectra were recorded using an FT-NMR spectrometer Bruker Avance III AV400 (400 MHz) with HMDS as an internal standard. Samples with a mass of 10 mg were dissolved in CDCl_3_. Chemical shifts were determined by the residual non-deuterated chloroform signal.

Analysis of the molecular weight of the polymers was performed using gel-permeation chromatography on the instrument Agilent Technologies 1260 Infinity with a refractive index detector, GPC/SEC – styrogel column, length 300 mm, internal diameter 7.5 mm, eluent (CHCl_3_) rate 1 mL/s, calibration according to the polystyrene standards known molecular weight.

Thermal analysis was performed using a DSC 204 F1 Phoenix (NETZSCH) at a heating rate 10 °С/min with aluminum pans (the lid was manually drilled to ensure the access of argon). The DSC instrument was first calibrated with an indium standard. Measurements were carried out under an inert argon (or air) atmosphere at a flow rate of 50 mL/min. Approximately 1 mg of virgin or oxidized sample was heated from 25 °С up to 250 °С.
